# Where eagles soar: Fine‐resolution tracking reveals the spatiotemporal use of differential soaring modes in a large raptor

**DOI:** 10.1002/ece3.4189

**Published:** 2018-06-11

**Authors:** Megan Murgatroyd, Theoni Photopoulou, Les G. Underhill, Willem Bouten, Arjun Amar

**Affiliations:** ^1^ FitzPatrick Institute of African Ornithology Department of Biological Sciences University of Cape Town Cape Town South Africa; ^2^ Animal Demography Unit Department of Biological Sciences University of Cape Town Cape Town South Africa; ^3^ Centre for Statistics in Ecology Environment and Conservation Department of Statistical Sciences University of Cape Town Cape Town South Africa; ^4^ Department of Zoology Institute for Coastal and Marine Research Nelson Mandela University Port Elizabeth South Africa; ^5^ Institute for Biodiversity and Ecosystem Dynamics University of Amsterdam Amsterdam Netherlands

**Keywords:** behavior classification, collision risk, energy landscape, flight, predictive modeling, random forest, soaring, uplift

## Abstract

Unlike smaller raptors, which can readily use flapping flight, large raptors are mainly restricted to soaring flight due to energetic constraints. Soaring comprises of two main strategies: thermal and orographic soaring. These soaring strategies are driven by discrete uplift sources determined by the underlying topography and meteorological conditions in an area. High‐resolution GPS tracking of raptor flight allows the identification of these flight strategies and interpretation of the spatiotemporal occurrence of thermal and orographic soaring. In this study, we develop methods to identify soaring flight behaviors from high‐resolution GPS tracking data of Verreaux’s eagle *Aquila verreauxii* and analyze these data to understand the conditions that promote the use of thermal and orographic soaring. We use these findings to predict the use of soaring flight both spatially (across the landscape) and temporally (throughout the year) in two topographically contrasting regions in South Africa. We found that topography is important in determining the occurrence of soaring flight and that thermal soaring occurs in relatively flat areas which are likely to have good thermal uplift availability. The predicted use of orographic soaring was predominately determined by terrain slope. Contrary to our expectations, the topography and meteorology of eagle territories in the Sandveld promoted the use of soaring flight to a greater extent than in territories in the more mountainous Cederberg region. Spatiotemporal mapping of predicted flight behaviors can broaden our understanding of how large raptors like the Verreaux’s eagle use their habitat and how that links to energetics (as the preferential use of areas that maximize net energy gain is expected), reproductive success, and ultimately population dynamics. Understanding the fine‐scale landscape use and environmental drivers of raptor flight can also help to predict and mitigate potential detrimental effects of anthropogenic developments, such as mortality via collision with wind turbines.

## 1 INTRODUCTION

The physical environment influences the energetic cost of animal locomotion and is known to modulate animal movement (Ganskopp, Cruz, & Johnson, 2000; Shepard et al., 2013; Spaar & Bruderer, 1996; Wall, Douglas‐Hamilton, & Vollrath, 2006; Wilson, Quintana, & Hobson, 2011). For soaring birds, the route, method, and cost of flight are greatly affected by the availability of uplift, which provides a harvestable supply of energy for movement (Katzner et al., 2015; Lanzone et al., 2012). Uplift used by large soaring birds can be broadly classified into two categories: (a) thermal uplift, which is driven by solar radiation heating the ground and warming the nearby air, thus creating rising columns of warm air (Akos, Nagy, Leven, & Vicsek, 2010), referred to as “thermals,” and (b) orographic uplift, which is upward air movement generated by wind deflected over topographic features. Birds exploit thermals by circling inside the upward‐moving air columns to gain altitude. Large soaring birds rely on thermals for uplift in flat terrain. Owing to the dependence of thermals on solar heating of the land surface and their dissipation by strong winds, thermals are spatiotemporally variable (Shepard & Lambertucci, 2013). In contrast, orographic uplift concentrates along ridgelines and is thought to provide favorable conditions for the long‐distance autumn migration of large soaring birds (Brandes & Ombalski, 2004; Bohrer et al., 2012. But see: Duerr et al., 2012, 2015; Katzner et al., 2015). Mountainous areas are usually associated with high uplift availability, owing to their ability to generate orographic uplift even in low‐wind conditions or cool temperatures (Shepard & Lambertucci, 2013).

Landscape features can determine spatial uplift availability at a fine scale, while weather conditions can modulate temporal changes in uplift availability (Dodge et al., 2014; Duerr et al., 2015; Shamoun‐Baranes et al., 2006; Spaar & Bruderer, 1996; Treep et al., 2016; Vansteelant, Bouten et al., 2014; Vansteelant, Verhelst et al., 2014). For example, when wind speeds increase, golden eagles *Aquila chrysaetos* shift flight strategies from thermal soaring to orographic soaring to reduce the energetic costs of migration (Lanzone et al., 2012). The altitude gained via either uplift type is a form of potential energy that reduces the need for powered flight providing calorific savings (Pennycuick, 2008). Therefore, the preferential use of areas that maximize net energy gain is expected. Although there is good theoretical understanding of uplift availability and its use for soaring (Bohrer et al., 2012; Pennycuick, 2008), empirical studies using modern technology to investigate whether birds utilize uplift in line with these expectations are only now emerging (Katzner et al., 2015; Lanzone et al., 2012; Péron et al., 2017; Shepard, Lambertucci, Vallmitjana, & Wilson, 2011; Sherub, Bohrer, Wikelski, & Weinzierl, 2016).

Recent advances in technology are increasingly allowing the identification of distinct movement behaviors in free‐living animals (Kays, Crofoot, Jetz, & Wikelski, 2015). Given these technological advances and the discrete nature of uplift, we are now in a position to model the spatiotemporal probability of uplift‐induced flight behaviors or availability of uplift as “energy landscapes” for soaring species (Shepard et al., 2013; Wilson et al., 2011). This advance will be important for a number of applications; for example, the ability to predict the distribution and probability of locomotory behaviors will be critical in mitigating anthropogenic risks associated with specific behaviors (Camacho, Palacios, Sáez, Sánchez, & Potti, 2014; Colchero et al., 2011; Péron et al., 2017; Reid, Krüger, Whitfield, & Amar, 2015). Values of thermal uplift potential have been shown to be useful in predicting flight altitude, and thus wind turbine collision risk, for Andean condors *Vultur gryphus* and griffon vultures *Gyos fulvus,* which predominately rely on thermal uplift for low‐energy flight and to a lesser extent for golden eagles, which switch between the use of orographic and thermal uplift (Péron et al., 2017).

Landscape features that promote or limit certain movement strategies can affect the energetic cost of locomotion, which are reflected in the “energy landscape” (Shepard et al., 2013; Wilson et al., 2011). At a broad scale, the energy landscape is likely to have implications for species distributions, and at a finer scale, it might impact on individual fitness or breeding performance (Shepard et al., 2011; Weimerskirch, Louzao, de Grissac, & Delord, 2012). Breeding performance has frequently been investigated in relation to factors such as food availability and meteorological conditions (Bosch, Martínez, Calvo, Zuberogoitia, & Jiménez‐Franco, 2014; McDonald, Olsen, & Cockburn, 2004; Millon, Arroyo, & Bretagnolle, 2008; Steenhof, Kochert, & Mcdonald, 1997), while links between the energy landscape and breeding performance have received little attention. Thus, a better understanding of soaring flight behaviors and their occurrence in the landscape could provide insights into the conditions under which large raptor species will suffer costs or reap benefits (Shepard & Lambertucci, 2013).

In this study, we identified behavioral states of a large soaring raptor; the Verreaux’s eagle *Aquila verreauxii,* using high‐resolution GPS tracking data collected in the Western Cape, South Africa. We examined the topographic and meteorological correlates of the two uplift‐assisted flight modes (thermal soaring and orographic soaring). Following this, we use these relationships to predict the probability of thermal and orographic soaring within known active eagle territories in our study area, which are presumed to reflect the local uplift availability. Finally, we compare the predicted use of soaring flights throughout the year between territories in two topographically contrasting regions (Cederberg and Sandveld) which are known to have dissimilar breeding performance (Murgatroyd, Underhill, Rodrigues, & Amar, 2016).

Based on the current knowledge of uplift availability and the effects of weather on soaring behavior in raptors, we predicted that (a) thermal soaring is correlated with warm still weather (favoring the formation of thermals) and flat topography; (b) orographic soaring is correlated with windy weather conditions and mountainous terrain slopes; (c) thermal soaring potential is greater in the Sandveld than in the Cederberg owing to generally flatter topography; (d) orographic soaring potential is greater in the Cederberg than in the Sandveld driven by mountainous topography and both areas are likely to be subject to seasonally fluctuating patterns in soaring flight potential; and finally, (e) the total soaring (orographic and thermal) potential is expected to be greater in the Cederberg than in the Sandveld as mountains are thought to represent optimal habitat for this species.

## 2 METHODS

### 2.1 Study area and model species

**Figure 1:**
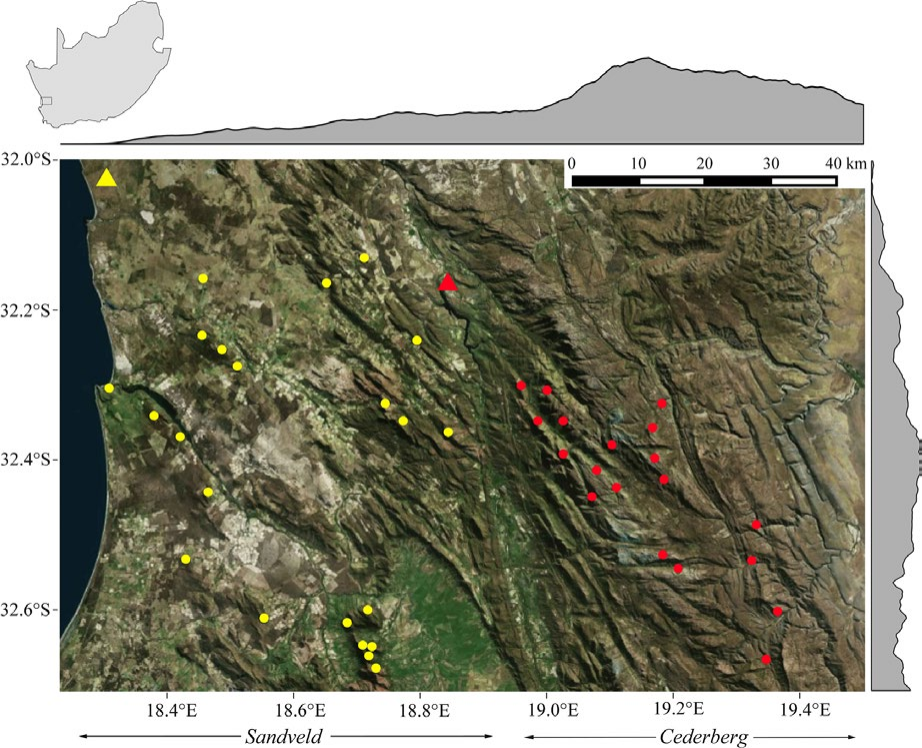
Study areas in the Western Cape, South Africa, with Verreaux’s eagle nests in the Sandveld (yellow dots) and the Cederberg (red dots). Meteorological data used in this study were derived from South African Weather Services stations at Lambert’s Bay (yellow triangle) and Clanwilliam (red triangle). Gray altitude profile shows mean altitude change throughout the region

This study was carried out in the Cederberg Mountains and the Sandveld region in the Western Cape, South Africa. Both areas experience hot dry summers and cool wet winters. The Cederberg forms the northern end of the Cape Fold Mountains, and the elevation ranges from *c*. 150 to 2,027 m. This study area is dominated by natural fynbos vegetation and is largely protected by provincial conservation authority, CapeNature (Maree & Vromans, 2010). Topography in the adjacent Sandveld is much flatter, with elevation ranging from sea level to *c. *1,000 m (Figure 1). Nests in this region are mostly located on isolated rocky outcrops interspersed through relatively flat plains. There is little formal conservation in the Sandveld, which is consequently highly fragmented by agriculture (Franke, Steyn, Ranger, & Haverkort, 2011; Heydenrych, 1993). Nevertheless, this region maintains an important population of Verreaux’s eagles characterized by high annual breeding productivity (Murgatroyd, Underhill, Rodrigues et al., 2016).

### 2.2 GPS data

We caught five adult Verreaux’s eagles close to known nest sites between April 2012 and April 2013 in the Sandveld (*n = *3) and the Cederberg (*n = *2). We used backpack‐style harnesses made from 0.55” tubular Teflon Ribbon^®^ (Bally Ribbon Mills, Bally, PA) to equip each eagle with a high‐resolution UvA‐BiTS GPS logger (University of Amsterdam Bird Tracking System; Bouten, Baaij, Shamoun‐Baranes, & Camphuysen, 2013; Murgatroyd, Underhill, Bouten, & Amar, 2016). The tags recorded a 3D position every 2 min on average during daylight hours. Additionally, the GPS loggers recorded an hour or more of high‐resolution data each day, depending on the battery voltage and the availability of solar charge. The high‐resolution segments are the focus of this study, which provide fixes once every 3 s, although due to occasional delays in making a GPS fix, we include up to 6 s as “high resolution.” (Supporting information Figure S1).

We carried out all data analyses in R version 3.1.2 (R Core Team 2014). Supporting information Figure S2 provides a summary of all analysis steps and relevant sample sizes. Our analysis of soaring behavior involved three main steps: (a) We classified segments of track from three eagles in terms of flight behavior (see Behavioral classification), (b) we used these classified flight behaviors as the response variable in a regression model to try and explain different behaviors using environmental and topographic covariates (see Statistical analysis of flight behavior), and (c) we used the regression model to predict the probability of thermal or orographic soaring across 37 known eagle territories in the study area under 100 different weather condition scenarios each month for each eagle territory (see Spatiotemporal predictions of soaring flight). Each of these steps is described in detail below.

### 2.3 Behavioral classification

We used GPS data from days that featured at least some locations sampled at high resolution for the behavioral classification. Although we knew prior to making behavioral classifications that not all lower resolution data could be accurately classified, we were unsure what temporal resolution cutoff point would be necessary to obtain accurate classifications. We therefore initially used all of the data from days that contained bouts of high‐resolution locations (3–6 s). This meant that some low‐resolution data were included in the sample to be classified and we later filtered these out appropriately (see filtering of data with sampling intervals ≤6 s).

We chose four behavioral categories to identify within the tracking data: perched (little change in altitude or horizontal location), gliding (flying and losing altitude), orographic soaring (flying in a roughly straight trajectory without losing altitude) or thermal soaring (spiral‐like flying with increasing altitude and relatively little horizontal movement). These broadly describe the types of flight behavior that Verreaux’s eagles regularly engage in, including one nonflight behavior. We classified each GPS point as belonging to one of the four behavioral categories, based on features extracted from all data points within a window extending 45 s either side of the location being considered, referred to as a “track segment.” This was done in order to capture the context of each point along the movement track, instead of considering it in isolation, because this method does not formally acknowledge the time series nature of the data. In total, this gives a 90‐s window over which to manually classify flight behavior during training (see below for more details on the classification method). This was done to capture the behavior that often has a short duration: thermal soaring. Depending on the strength and the height of the thermal, birds can exploit the available uplift for just a few seconds or over a minute. The “zoomed out” view that the 90‐s window provides makes it easier to identify the flight behavior during the training step of the classification.

We used a random forest ensemble learning method for classification, to predict the behavior associated with each point, due to its efficiency and low error rate (Breiman, 2001). We implemented the supervised learning version of the algorithm as visualization of GPS tracks and their movement parameters readily allow for manual classification of sections into behavioral categories to form a training set (Supporting information Figure S3). Once manual classification of a portion of the data by a human is complete, the random forest model can be used to predict behavior for unseen data. For the training set, we drew a random sample of locations representing 10% of all eagle tracking days that included some high‐resolution data. We sampled 10% of tracking days from each tagged bird because each bird was tracked for a different number of days.

We extracted six movement features for each track segment and used them in the random forest as predictors. First, (a) we calculated the mean of the three‐dimensional instantaneous speed. Second, we fitted linear regression models to altitude above sea level as a function of time and altitude above ground level as a function of altitude above sea level. We extracted a total of four features from these two linear regression models; (b) the rate of change of altitude above sea level; (c) the rate of change of altitude above ground level; (d) the squared correlation coefficient *R* ^2^ (equal to the correlation coefficient *r* ^2^ as it comes from a simple linear model, i.e., one with a single covariate) for the linear regression model of altitude above sea level against time; and (e) the *R* ^2^ (as above) for the linear regression model of altitude above ground level against altitude above sea level. Lastly, the “spectrum” function in R (R Core Team 2014, Bloomfield 1976) was used to calculate a periodogram and estimate the spectral density of the time series of directions within the track segment. Spectral density estimation is a way of breaking down a pattern in a time series that displays sinusoidal periodicity or cyclical dynamics (such as direction during thermalling) by calculating the contribution of individual frequencies (the number of times an event repeats or cycles within a unit of time, here compass bearings in degrees within a track segment) to the observed pattern. In other words, it is a way of identifying the different frequencies that make up the overall signal and the importance of each one (Chatfield, 2003). The dominant frequency of a time series is the one that contributes the most. One of the flight behaviors of interest, thermal soaring, is characterized by cyclical patterns so for the sixth movement feature (f) we extracted the dominant frequency in the time series of direction. Periodicity in direction results in high dominant frequency, whereas traveling in a relatively straight trajectory results in a low dominant frequency. These six movement features reflect distinctive characteristics of the different behaviors (see Table 1 for a list describing each feature and the rationale for using it). For example, a high correspondence between altitude above sea level and altitude above ground level, as well as regular cyclical changes in direction, was a good predictor of thermal soaring (see Supporting information Figure S3 for examples of the movement characteristics typical of orographic and thermal soaring).

**Table 1:** Description and rationale of the features used in the random forest algorithm to predict flight behaviors based on each 90‐s track segment

Feature number in text	Feature description	Rationale
a)	Mean of the three‐dimensional instantaneous speed (m/s)	Speed varies between behaviors; for example, perching is associated with low or zero speed, so this feature will help identify periods of perching.
b)	Rate of change of altitude above sea level (m/s)	During thermal soaring, birds gain altitude steadily compared to other behaviors, whereas in gliding birds lose altitude steadily.
c)	Rate of change of altitude above ground level (m/s)	During orographic soaring, birds can more or less maintain altitude above ground compared to thermal soaring where they gain altitude relative to the ground.
d)	The proportion of variation in altitude above sea explained by time (*R* ^2^ in a linear regression model of time against altitude above sea level)	If there is a reliable straight‐line relationship between time and altitude above sea level (large *R* ^2^), this suggests that the bird is steadily gaining or losing altitude in absolute terms but may not be gaining altitude relative to the ground. This might be expected to happen during orographic soaring.
e)	The proportion of variation in altitude above ground explained by altitude above sea level (*R* ^2^ in a linear regression model of altitude above sea level against altitude above ground level)	If there is a reliable straight‐line relationship between altitude above sea level and altitude above ground level (large *R* ^2^), this suggests that the bird is steadily gaining or losing altitude relative to the ground, as happens in thermal soaring or gliding.
f)	Spectral density of the time series of directions (compass bearing in degrees)	A high spectral density means that there were repeating sequences of directions within a track segment, like those resulting from the circular flight path of thermalling birds. A low spectral density suggests that there were not repeating sequences of directions and that the flight path was straight or erratic.

These features were extracted for all days with at least some high‐resolution data. We flagged and removed data‐poor segments (those with fewer than five locations and segments without any 3‐s data) because they would have potentially unreliable predictions attached to them due to low data quality. Within the remaining segments, only data with sampling intervals ≤6 s were extracted for further analysis, as these data were thought to have the most reliable behavioral classifications. The rationale for keeping data sampled at ≤6 s is as follows: (a) Due to tag capabilities and schedules, this dataset was in fact largely (>99%) data sampled at 3 to 4s intervals, so we did not lose large amounts of data by doing this and (b) it was not possible to reliably resolve behaviors, particularly thermalling, at intervals more than or equal to 10 s (due to the loss of sinusoidal periodicity signals), and no data were collected at 7 to 9s intervals. We further subsampled the data into those collected between 11:00 and 15:00 SAST daily, coinciding with when eagles are most active (Murgatroyd, Underhill, Bouten et al., 2016) and this is also the period when most high‐resolution data were collected. Owing to this subsampling, data from only three of the five eagles were reliably classified and used in further analyses.

### 2.4 Meteorological and topographic variables

We derived topographic variables (elevation [m], slope [°], and aspect [°]) from a 30m‐resolution Shuttle Radar Topography Mission (SRTM) digital elevation model (DEM) using the “raster” package (Hijmans 2015). The South African Weather Services (SAWS) provided meteorological variables (wind direction [°], wind speed [m/s], and temperature [°C]) at hourly intervals from 2012 to 2013. For eagles tracked in the Cederberg, we used meteorological data recorded at Clanwilliam (32.1760S, 18.8880E), and for eagles tracked in the Sandveld, we used data recorded at Lambert’s Bay (32.0350S, 18.3320E), which are *c*. 30 km from the nest sites of the tracked eagles (Figure 1). Although this is spatially coarse data, we expected to detect some correlations between flight behavior and weather variables due to the temporal resolution of the data.

The angle of incidence (“*v*”; °) between the topographic aspect and wind direction was calculated to account for the expected relationship between these variables on windward and leeward slopes. Values can range from 0 to 180°, whereby high values correspond with the wind directly hitting the slope (e.g., a south‐facing slope and wind coming from the south). *v* equal to 90° would be equivalent to wind perpendicular to the topographic aspect (e.g., a south‐facing slope and wind coming from either the east or the west). The lowest values correspond with the lowest potential to generate orographic uplift, where the wind is coming from directly behind a slope. The R function for *v* is given in Supporting information Appendix S1. Wind direction and aspect were not included in the models individually owing to the understanding that their interaction is more important than their singular effects in driving orographic uplift (Bohrer et al., 2012).

To calculate the sun exposure on the terrain, we used a hill shading equation for each grid square of the DEM, dependent on date and time (Burrough & McDonell, 1998), where all variables were entered as radians and hill shade values of less than 0 were zeroed. The azimuth (*a*) and zenith (*z*) of the sun were extracted using the “insol” package (Corripio, 2015). Slope (θ) and aspect (β) values were extracted from the DEM. High values of hill shade indicate high sun exposure on the terrain.

Hill shade=255(Cos(z)Cos(θ)+Sin(z)Sin(θ)Cos(α−β))

### 2.5 Statistical analysis of flight behavior

We excluded all points where eagles were classified as perched to focus only on flight behavior. GPS locations classified as “gliding” were included in the response variable to represent the potential environment in which flight occurs, but owing to the fact that gliding occurs largely independently of the underlying topography, it is dependent on altitude gained in prior soaring flights and could be assisted by occasional wing beating (Pennycuick, 2008), gliding probabilities could not be tested in the same manner as soaring. The relationships between soaring flight behaviors (thermal and orographic soaring) and meteorological and topographic variables were explored using two generalized linear models with binomial response variables (i.e., thermal model: 1 = thermal soaring; 0 =  other flying points; orographic model: 1 = orographic soaring; 0 = other flying points) and logit link functions. Elevation and slope were additionally included in the models as quadratic variables to account for potential nonlinear relationships between uplift behaviors and topography, such as the good potential for the formation of thermals through solar heating of contrasting land surfaces (i.e., low‐altitude flat areas and sun‐exposed rocky slopes) (Shamoun‐Baranes, Leshem, Yom‐Tov, & Liechti, 2003). To examine which variables best explained thermal and orographic soaring, a model selection approach was implemented using Akaike’s information criterion (AIC). We ranked models according to their AIC score using the “MuMIn” package (Barton, 2014), and where there was not a clear top model (Akaike’s weight > 0.9), we used model averaging across all models with a delta‐AIC of <2. Area under the curve (AUC) value of a receiver operating characteristic (ROC) was used to assess the final accuracy of the models analyzing flight behavior (Robin et al., 2011; Swets, 1988).

### 2.6 Spatiotemporal predictions of soaring flight

We defined a territory as a 3km buffer around known nest sites, based on previous home range estimates (Murgatroyd, Underhill, Bouten et al., 2016). All known active territories previously monitored for breeding (Murgatroyd, Underhill, Rodrigues et al., 2016) were included, except for one in the Sandveld (where there was more than 50% overlap of the territory buffer with the sea) (Cederberg *n = *19; Sandveld *n = *18). We extracted topographic data from the DEM for all grid squares within each territory. The availability of uplift is expected to change throughout the day and throughout the year with seasonal changes in weather patterns (Duerr et al., 2015). Therefore, we used stratified random sampling of the weather conditions to represent monthly conditions in each area (see below). The South African Weather Services (SAWS) provided meteorological data from weather stations located at Clanwilliam (for Cederberg territories) and Lambert’s Bay (for Sandveld territories). Data used were recorded at hourly intervals from 07:00 to 19:00 SAST between 2012 and 2013, which are inclusive of the GPS tracking period. After the exclusion of missing weather values, there were 8,768 valid weather scenarios, which each include temperature, wind direction, and wind speed, in the Sandveld and 8,627 in the Cederberg over the 2 years, from which we used 100 random samples per month (January–December, irrespective of year) for each area.

Using the inverse logit probability of parameter estimates for the variables in the final GLMs, the probability of thermal and orographic soaring was predicted in each grid cell of all eagle territories (with resolution taken from the original 30 m DEM) for the 100 meteorological scenarios per month. Following this, we calculated the mean probability of thermal and orographic soaring for each month of the year. To assess the effects of seasonal changes on the overall use of soaring modes, the probability of thermal and orographic soaring was combined to obtain an estimate of the total potential for soaring flight. Welch’s two‐sampled *t*‐tests were used to test for significant differences in the predicted use of soaring between the study areas on a monthly basis.

## 3 RESULTS

### 3.1 Behavioral classification

More than 160,000 GPS locations collected over 130 days from three eagles were reliably classified as one of the four behaviors (Supporting information Table S1). In random forest models, the data are subsampled many times during the training phase, and a decision tree is built for each subsample. The overall prediction error is measured as the mean prediction error of each subsample using only the trees that were not trained on that subsample. This is called the “out‐of‐bag” error rate (OOB). In our case, the overall OOB of the flight behavior classification model was 9%. The class with the highest classification error was orographic soaring (42%), followed by gliding (19%) and thermal soaring (18%), while perching (<1%) had the lowest classification error (Supporting information Table S2). Following temporal subsetting and exclusion of perched points, 42,883 GPS locations were classified as flying and used in further analysis (Supporting information Table S3).

### 3.2 Analysis of soaring flight behavior

Two top models (ΔAIC < 2) explained thermal soaring, and we used model averaging over these to obtain model‐averaged estimates for predicting the use of thermal soaring (Supporting information Table S4). The final model included all of the variables that were considered in initial models. The AUC value for this model was 0.615. Thermal soaring showed a statistically significant quadratic correlation with elevation (Table 2, Supporting information Figure S4). Below *c. *750 m, variations in elevation had little effect on the probability of thermal soaring, and above this height, the probability decreased. There was a significant quadratic relationship between thermal soaring and slope (Table 2, Supporting information Figure S4). The probability of thermal soaring was the highest in flat areas and on particularly steep slopes, while intermediate slopes were used less for this behavior. There was a positive correlation between hill shade and thermal soaring. Likewise, there was a positive but statistically insignificant correlation between temperature and thermal soaring (Table 2, Supporting information Figure S4). Thermal soaring showed a negative correlation with wind speed (Table 2, Supporting information Figure S4) and the angle of incidence between the topographic aspect and the wind direction (Table 2). Thus, thermal soaring occurs most often over low‐elevation terrain, on flat ground, and on steep slopes, when sun exposure and temperature were greater and on leeward sides of slopes or in conditions of low wind.

**Table 2:** Model‐averaged estimates predicting the probability of thermal soaring by Verreaux’s eagles

	Estimate	*SE*	*z*‐value	*p*	Confidence intervals	
					2.5%	97.5%
(Intercept)	−0.591	0.081	7.26	<0.005	−0.75	−0.43
Elevation	4.82 × 10^−4^	1.10 × 10^−4^	4.37	<0.005	2.66 × 10^−4^	6.98 × 10^−4^
Elevation^2^	−6.15 × 10^−7^	5.56 × 10^−8^	11.06	<0.005	−7.24 × 10^−7^	−5.06 × 10^−7^
Slope	−0.035	0.003	12.62	<0.005	−0.04	−0.03
Slope^2^	0.001	4.96 × 10^−5^	11.48	<0.005	4.72 × 10^−4^	6.66 × 10^−4^
Wind speed	−0.091	0.010	9.00	<0.005	−0.11	−0.07
v	−0.001	2.03 × 10^−4^	6.03	<0.005	−1.62 × 10^−3^	−8.27 × 10^−4^
hs	4.19 × 10^−3^	3.19 × 10^−4^	13.11	<0.005	3.56 × 10^−3^	4.81 × 10^−3^
Temperature	1.72 × 10^−4^	0.001	0.15	0.88	−3.60 × 10^−3^	4.84 × 10^−3^

*Note*. hs, hill shading (representing sun exposure); v, angle of incidence between aspect and wind direction.

After model averaging over four top models (Supporting information Table S5), the final model for orographic soaring also included all variables. The AUC value for this model was 0.617. Orographic soaring showed a significant positive correlation with slope angle and wind speed (Table 3, Supporting information Figure S4). Orographic soaring showed a quadratic correlation with elevation (Table 3, Supporting information Figure S4). Positive but insignificant trends were shown between the angle of incidence and orographic soaring (Supporting information Figure S4, Table 3). There was a negative correlation with temperature and hill shade although these were also insignificant. Thus, orographic soaring occurs most often on steep‐sided slopes at high elevations, and in windy conditions, which produces strong orographic uplift.

**Table 3:** Model‐averaged estimates predicting the probability of orographic soaring by Verreaux’s eagles

	Estimate	*SE*	*z*‐value	*p*	Confidence intervals	
					2.5%	97.5%
(Intercept)	−1.29	0.091	14.26	<0.005	−1.47	−1.12
Elevation	−9.70 × 10^−4^	1.21 × 10^−4^	8.01	<0.005	−1.21 × 10^−3^	−7.33 × 10^−4^
Elevation^2^	4.70 × 10^−7^	5.65 × 10^−8^	8.32	<0.005	3.59 × 10^−7^	5.80E ×10^−7^
Slope	0.034	0.003	11.62	<0.005	0.03	0.04
Slope^2^	−1.06 × 10^−4^	4.99 × 10^−5^	2.13	<0.05	−2.04 × 10^−4^	−8.25 × 10^−6^
Wind speed	0.047	0.010	4.47	<0.005	0.03	0.07
v	3.26 × 10^−4^	2.48 × 10^−4^	1.32	0.19	−6.90 × 10^−6^	8.17 × 10^−4^
hs	−2.28 × 10^−5^	1.33 × 10^−4^	0.17	0.86	−7.39 × 10^−4^	4.57 × 10^−4^
Temperature	−2.29 × 10^−3^	2.51 × 10^−3^	1.15	0.25	−8.10 × 10^−3^	4.22 × 10^−4^

*Note*. hs, hill shading (representing sun exposure); v, angle of incidence between aspect and wind direction.

### 3.3 Predicting uplift availability

**Figure 2:**
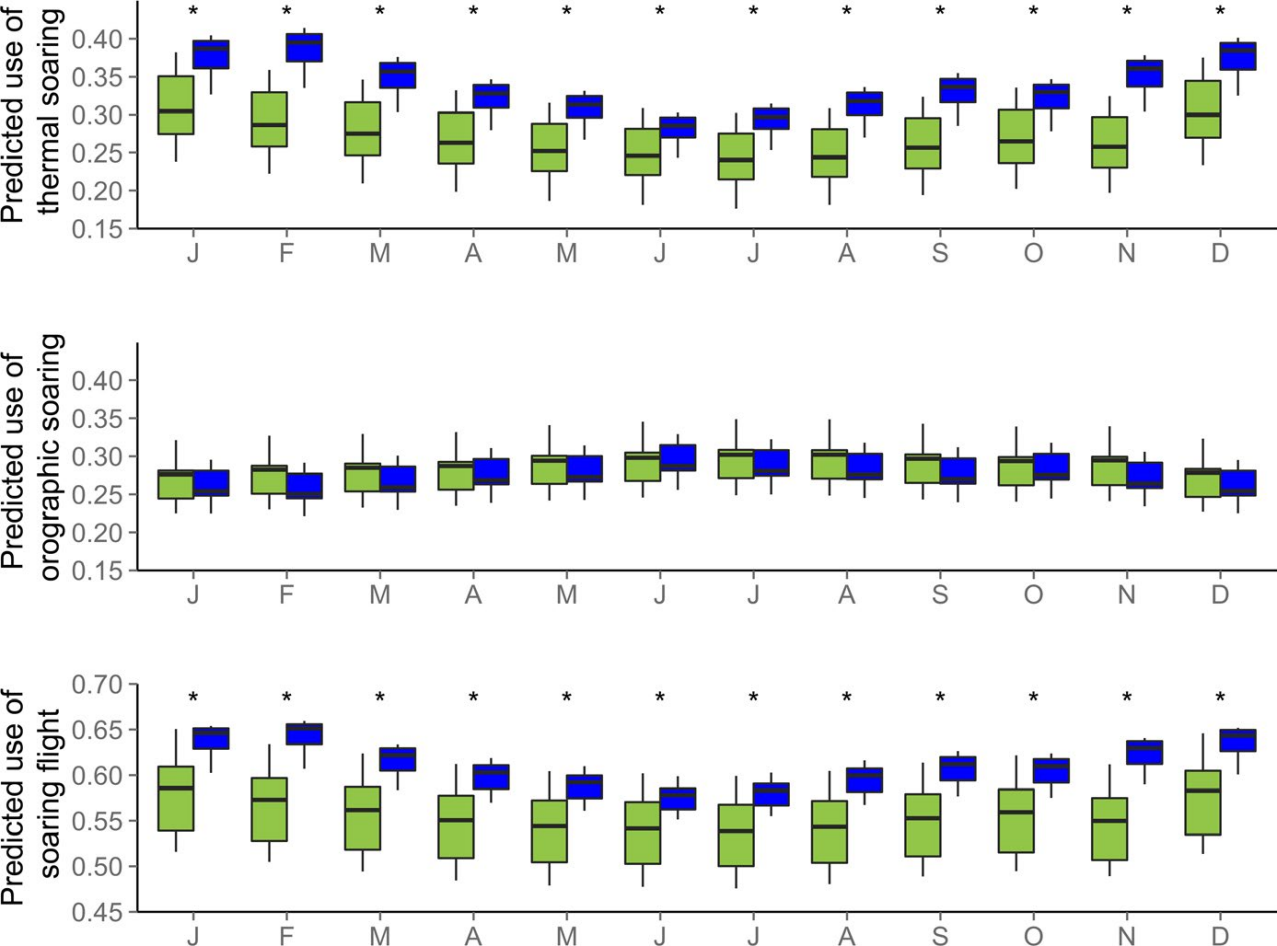
Predicted use of thermal and orographic soaring and their combined totals in Verreaux’s eagle territories in the Cederberg (green, *n = *19) and the Sandveld (blue, *n = *18) throughout the year. *significant monthly differences between study areas

The predicted use of soaring flight varied throughout the year (Figure 2). The predicted use of thermal soaring was generally highest outside of the breeding season (during summer months). The predicted use of orographic soaring was highest during the breeding season (winter months).

**Figure 3:**
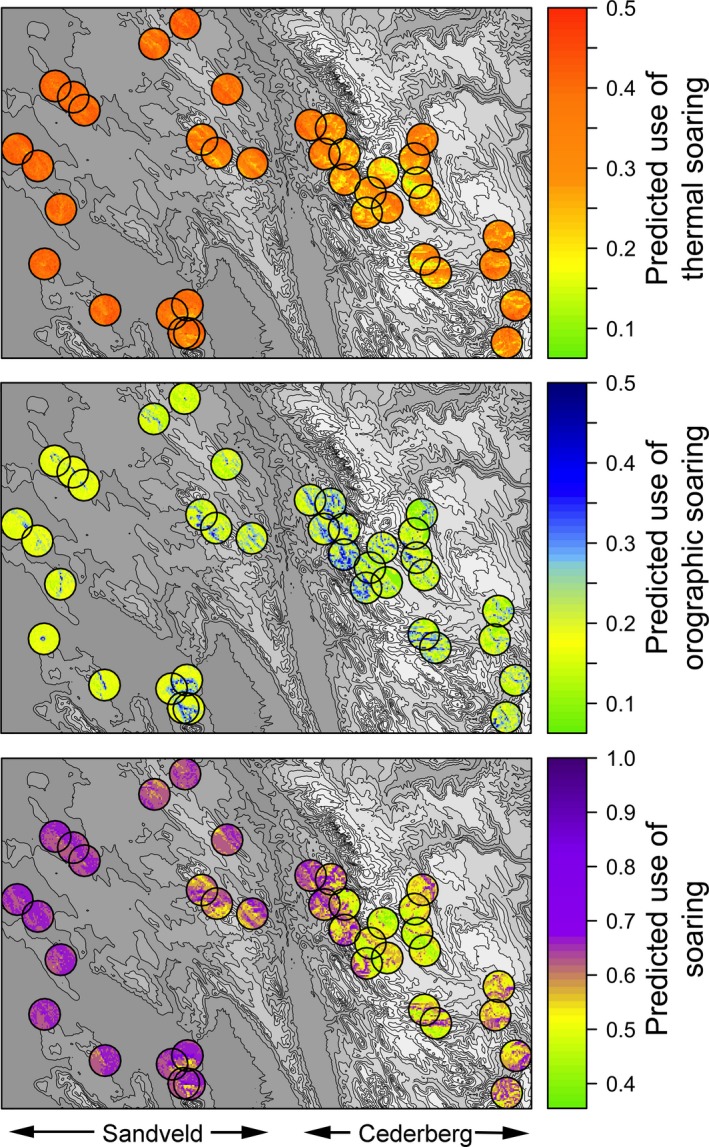
Predicted use of thermal, orographic, and both soaring flight modes in Verreaux’s eagle territories (3‐km‐radius circular buffers around known nest sites) in the Cederberg and the Sandveld for a random weather scenario in June. Digital elevation model (gray shade; 0–2,000 masl) demonstrates topography

The predicted use of thermal soaring was significantly higher during all months for territories in the Sandveld compared to those in the Cederberg (Figure 2, Supporting information Table S6). The mean predicted use of orographic soaring was higher in territories in the Cederberg than in the Sandveld, although this difference was insignificant across months. The total predicted use of soaring flight modes was significantly greater in territories in the Sandveld than in the Cederberg throughout the year (Figure 2, Supporting information Table S6). Between‐territory variations in soaring opportunities were greater in the Cederberg than in the Sandveld, evidenced by the larger error bars (Figure 2). The predicted use of soaring flight was illustrated for all study nests in the Cederberg and the Sandveld for a random weather scenario (Figure 3).

## 4 DISCUSSION

High‐resolution GPS tracking data from Verreaux’s eagles allowed the identification of fine‐scale responses to the spatiotemporally variable environment in which flight occurs. We found that the use of contrasting soaring modes followed patterns that can be explained by an understanding of the formation of thermal and orographic uplift (Bohrer et al., 2012; Brandes & Ombalski, 2004; Katzner et al., 2015), allowing the use of soaring flight modes to be predicted in spatially and temporally heterogeneous environments.

We found that topographic and meteorological variables contributed to determining the method of soaring. In line with our prediction (a), thermal soaring usually occurred at lower elevations and over flat topography, although there was additional evidence that eagles also use steep slopes for thermal soaring opportunities. Rocky mountain slopes facing the sun have the ability to heat up fast, and this can result in the formation of good thermal uplift (Shamoun‐Baranes et al., 2003). High wind speed causes turbulence that tends to break down or inhibit the formation of thermals (Bohrer et al., 2012; Shepard & Lambertucci, 2013), and this was evident in the selection for low wind speed and leeward locations for thermal soaring. In contrast to thermal soaring, eagles tend to use orographic soaring on the windward rather than the leeward side of a slope (Bohrer et al., 2012). In agreement with our second prediction (b) that orographic soaring favored windy steep slopes, our results showed some evidence that a high angle of incidence between terrain aspect and wind direction was preferred, which drives air movements over topographic features to create orographic uplift. However, the spatial resolution of wind direction data was not fine enough to adequately represent this variable given the potentially fine‐scale variability of wind direction against slopes and the coarse‐scale variability of the meteorological data that we used. The slope angle and wind speed both were positively correlated with orographic soaring, which are known to drive orographic uplift availability and strength (Bohrer et al., 2012).

Using these correlates of soaring flight, it was possible to predict the spatiotemporal probability of soaring and this might also reflect the predicted availability of thermal and orographic uplift across the landscape. In accordance with prediction (c), the probability of thermal soaring was higher in eagle territories in the Sandveld than in the Cederberg. This was consistent for every month of the year and was driven by the lower elevation and flatter topography in the Sandveld compared to the Cederberg. The probability of orographic soaring was greater in some individual territories in the Cederberg than in the Sandveld. However, contrary to prediction (d), overall differences in the probability of orographic soaring between the two areas were not statistically significant.

The probability of both types of soaring flight was subject to seasonal fluctuations. Greater sun exposure on slopes and warm temperature induce the formation and strength of thermals and this was evident in the increase in probability of thermal soaring during austral summer months, outside of the breeding season. A greater use of thermal soaring in the summer season, compared to the winter, has been recorded in other large raptors (Nathan et al., 2012). Similarly, on a daily scale, both speed and altitude of migratory flight of soaring raptors increase during the hours around midday when stronger thermals develop (Mellone et al., 2012). Conversely, during the breeding season, the probability of orographic soaring tended to increase. These changes were not related to wind speed, which on average was not greater during the breeding season than outside of it, although the change in wind direction may have been important (Supporting information Table S7).

The consistently higher probability for thermal soaring in the Sandveld than in the Cederberg meant that the relatively flat Sandveld actually has greater total predicted use of soaring flight. This demonstrates that contrary to our expectations (prediction v), the flatter Sandveld area might actually present preferable flight habitat with more widespread uplift availability compared to the mountainous Cederberg area. However, this is only one measure of uplift usefulness to soaring birds. In addition to the predicted use of soaring flight, which might represent a measure of availability of uplift, uplift strength and the behaviors associated with each soaring flight type are relevant in determining usefulness to eagles. Uplift strength can determine the speed at which a soaring bird can gain altitude (Shepard et al., 2011; Spaar & Bruderer, 1996). Thermal uplift might be beneficial for cross‐country flying, but less useful for foraging, where eagles will need to maintain visibility of the ground (Péron et al., 2017; Shepard et al., 2011). Therefore, the relative usefulness that uplift plays in day‐to‐day behavior would be a useful topic for further investigation. If thermal soaring is more energetically beneficial than orographic soaring and there is better availability of this uplift type in the Sandveld, then these differences could contribute to the significantly higher breeding productivity of eagles in the Sandveld than in the Cederberg, a finding which is not yet fully understood (Murgatroyd, Underhill, Rodrigues et al., 2016). Higher energy costs will require greater prey availability and food intakes for them to be energetically plausible (Wilson et al., 2011); otherwise, a negative effect on population demography or individual fitness would be expected (Weimerskirch et al., 2012). Further research examining the relationship between breeding performance and the energy landscape on a year‐by‐year and nest‐by‐nest basis may shed light on this. It could also reveal a link between the interterritory variations in the predicted use of soaring flight in the Cederberg and the breeding performance of particularly productive and unproductive territories in the area (pers. obs).

Additional information on the relative energy requirements associated with each flight type would also benefit our understanding of the relative costs incurred by eagles inhabiting contrasting environments. Theory suggests that windier conditions incur greater metabolic costs of flight (Pennycuick, 1972) and such conditions favor orographic soaring flight. Therefore, eagles living in conditions suited to orographic soaring might incur greater energetic costs. Migration of golden eagles is faster and more energetically efficient when thermal soaring combined with gliding is used compared to orographic soaring (Duerr et al., 2012), and migratory flight has been positively correlated with weather conditions promoting thermal uplift, suggesting that eagles preferentially travel when conditions favor thermal soaring over orographic soaring (Duerr et al., 2015). Terrain ruggedness has been identified as an important determinant of increased heart rate in turkey vultures *Cathartes aura* due to its effect on decreasing the spatiotemporal predictability of uplift (Mandel, Bildstein, Bohrer, & Winkler, 2008), again potentially representing the cost associated with soaring in highly variable mountainous conditions. However, whether these advantages would apply equally to resident eagles ranging within their own territories is debatable.

A major limitation in this study was the coarse spatial resolution of meteorological data available, which is unlikely to sufficiently represent the fine‐scale responses of soaring raptors to environmental conditions. This may have reduced the strength of some correlations that were observed or underestimated the predictions for flight strategies primarily determined by meteorological factors. Although the trends we found were generally in agreement with our predictions, the results relating to flight responses to meteorological variables displayed larger confidence intervals and lower statistical significance than topographic variables (e.g., the role that temperature and wind direction play). Like aspect, the angle of incidence was expected to be significant in predicting the use of soaring flights, although the trends were in agreement with our predictions these findings did not reach statistical significance. Small‐scale spatial changes in airflow and the resultant uplift are not reflected in the meteorological data we used, particularly in mountainous terrain. To improve this in future applications of these methods, it will be important to obtain finer‐resolution meteorological data to capture the fine‐scale spatiotemporal soaring responses to environmental conditions.

Like many satellite tracking studies, ours suffers from a small sample size, in terms of both the number of individual birds that kept their devices on and the number of days for which those devices stayed on. Although we have made predictions of the use of soaring flight for all closely monitored eagle territories in the study area (37 in total), our results are based on three of the first five Verreaux’s eagles ever to be tracked. In addition, the three individuals were unequally represented in terms of days contributed to the final dataset used to make the predictions; however, all three birds were closely observed (by the lead author) for a period of 3 years during the course of the study and whose behavior was known to be typical of eagles in the respective areas. The individual that contributed the largest amount of data was an adult bird whose track was archetypal of what one would expect for a Verreaux’s eagle in the Cederberg Mountains. It remained within its expected territory for the duration of the tracking period (227 days including low‐resolution fixes) with few exploratory trips outside of that, and during this time, it made a single breeding attempt. We therefore feel confident that our dataset, though small, is representative of typical eagle ranging and flight behaviors in this area. On that account, one could say that what our results fall down on, in terms of the bulk of data, they gain in terms of novelty and original contribution for this species in this region. A larger sample size would undoubtedly increase our confidence in our results, but we see no reason for it to dramatically change the conclusions of the study.

This research adds to the growing body of work that uses onboard data loggers to test the underlying theory of soaring flight (Shepard, Ross, & Portugal, 2016; Sherub et al., 2016; Treep et al., 2016; Weinzierl et al., 2016). In addition to understanding the costs and benefits of inhabiting contrasting energy landscapes, improved knowledge of flight behaviors can be used for modeling potential anthropogenic risks, in particular the effects of wind turbines and associated energy infrastructure (McLeod, Whitfield, & McGrady, 2002; Reid et al., 2015). Large raptors are generally in decline in South Africa. In the Western Cape, severe drought is putting pressure on the agricultural sector, which eagles are sometimes in conflict with, and the growth of renewable energy developments is an emerging threat in terms of mortality through collisions with turbines and associated structures, particularly in favorable high‐wind habitats. This creates a strong need for a better understanding of how large raptors like the Verreaux’s eagle use their habitat and how that links to energetics, reproductive success, and ultimately population dynamics. This study, though limited by its small sample size, presents a glimpse into the flight behavior of Verreaux’s eagles in an area that has so far been a stronghold of the species in South Africa. It also lays the groundwork for more in‐depth studies on the impact of habitat and weather on landscape use by illustrating that fine‐scale flight behaviors can be better understood using widely available topographic and meteorological information. Thus, it will be important to extend this research to model and mitigate future risk in light of the proliferating wind energy industry globally.

## CONFLICT OF INTEREST

None declared

## AUTHOR CONTRIBUTIONS

MM carried out fieldwork, analyzed data, and wrote the manuscript. TP implemented the behavioral classifications and contributed to writing the manuscript. WB provided equipment and technical knowledge. AA and LGU helped conceptualize the study and contributed to data analysis. All authors reviewed and commented on drafts of this manuscript.

## DATA ACCESSIBILITY

Data are currently stored on the UvA‐BiTS database. This is user‐controlled owing to the sensitivity of the data, but data can be made available on request to the authors.
