# Supplementary material for: Where eagles soar: Fine‐resolution tracking reveals the spatiotemporal use of differential soaring modes in a large raptor
Source: Ecol Evol. 2018 Jun 11;8(13):6788–99. doi: 10.1002/ece3.4189 (PMC6053586; doi:10.1002/ece3.4189)
Supplement: Supplementary file 1 [file ECE3-8-6788-s001.docx]

# Supplementary Materials

Table S1. Summary of adult Verreaux’s eagle GPS tacking data collected in the Cederberg and the Sandveld, South Africa from 2012−2013, used for behavioural classification.

| Eagle id | Area | *n* unique days | *n* fixes |
| --- | --- | --- | --- |
| 721 | Cederberg | 4 | 9890 |
| 722 | Cederberg | 165 | 347330 |
| 723 | Sandveld | 7 | 15669 |
| 726 | Sandveld | 1 | 1329 |
| 727 | Sandveld | 1 | 1519 |

Table S2. Confusion matrix of behavioural classifications of Verreaux’s eagle tracking data, with class error. Abbreviations: t, thermal soaring; g, gliding; o, orographic soaring; p, perched.

|  |  | Predicted | | |  |  |
| --- | --- | --- | --- | --- | --- | --- |
|  |  | t | g | o | p | Class error |
| Observed | t | 1499 | 49 | 275 | 7 | 0.181 |
|  | g | 30 | 1922 | 288 | 155 | 0.198 |
|  | o | 275 | 340 | 1000 | 110 | 0.420 |
|  | p | 0 | 62 | 24 | 11845 | 0.007 |

Table S3. Summary of Verreaux’s eagle GPS data reliably classified into flight categories (g, gliding: o, orographic soaring; t, thermal soaring) and used in further analysis.

| Eagle id | Area | Date range | *n* unique days | *n* fixes | | |
| --- | --- | --- | --- | --- | --- | --- |
|  |  |  |  | g | o | t |
| 721 | Cederberg | 08/04/2012−14/04/2012 | 3 | 709 | 610 | 343 |
| 722 | Cederberg | 09/10/2012−20/03/2013 | 121 | 15887 | 10481 | 11243 |
| 723 | Sandveld | 26/08/2012−11/09/2012 | 6 | 1312 | 956 | 1342 |

Table S4. Models predicting thermal soaring by Verreaux’s eagles ranked by AIC value with change in AIC from top model < 2 (ΔAIC). Model parameters abbreviations: elev, elevation; elev^2^, quadratic term; slope, topographic slope; slope^2^, quadratic term; temp, temperature; w.speed, wind speed; v, angle of incidence between topographic aspect and wind direction (calculated in degrees); hs, hill shade. Other column abbreviations: df, degrees of freedom; logLik, log likelihood; *w*_i_, AIC weight.

| Model | df | logLik | ΔAIC | *w*_i_ |
| --- | --- | --- | --- | --- |
| elev+elev^2^+slope+slope^2^+w.speed+v+hs | 8 | -25478.41 | 0 | 0.723 |
| elev+elev^2^+slope+slope^2^+temp+w.speed+v+hs | 9 | -25478.36 | 1.92 | 0.277 |

Table S5. Models predicting orographic soaring by Verreaux’s eagles ranked by AIC value with change in AIC from top model < 2 (ΔAIC). Model parameters abbreviations: elev, elevation; elev^2^, quadratic term; slope, topographic slope; slope^2^, quadratic term; temp, temperature; w.speed, wind speed; v, angle of incidence between topographic aspect and wind direction (calculated in degrees); hs, hill shade. Other column abbreviations: df, degrees of freedom; logLik, log likelihood; *w*_i_, AIC weight

| Model | df | logLik | ΔAIC | *w*_i_ |
| --- | --- | --- | --- | --- |
| elev+elev^2^+slope+slope^2^+temp+w.speed+v | 8 | -24741.16 | 0 | 0.395 |
| elev+elev^2^+slope+slope^2^+w.speed+v | 7 | -24742.62 | 0.94 | 0.247 |
| elev+elev^2^+slope+slope^2^+temp+w.speed | 7 | -24742.86 | 1.41 | 0.195 |
| elev+elev^2^+slope+slope^2^+temp+w.speed+v+hs | 9 | -24741.05 | 1.79 | 0.162 |

Table S6. Monthly average (±SD) predicted (thermal, orographic and total) soaring availability in Verreaux’s eagle territories in the Cederberg (*n*=19) and the Sandveld (*n*=18) and the results from Welch’s two-sampled *t*-tests to identify significant differences in uplift opportunities per month between the study areas (bold).

|  | Cederberg | Sandveld | t | df | p |
| --- | --- | --- | --- | --- | --- |
| Thermal Soaring | |  |  |  |  |
| Jan | 0.31 ±0.04 | 0.38 ±0.03 | -5.78 | 29.16 | **<0.001** |
| Feb | 0.29 ±0.04 | 0.39 ±0.03 | -8.31 | 30.25 | **<0.001** |
| Mar | 0.28 ±0.04 | 0.35 ±0.02 | -6.35 | 29.07 | **<0.001** |
| Apr | 0.27 ±0.04 | 0.32 ±0.02 | -5.14 | 28.32 | **<0.001** |
| May | 0.25 ±0.04 | 0.31 ±0.02 | -5.21 | 28.09 | **<0.001** |
| Jun | 0.25 ±0.04 | 0.28 ±0.02 | -3.21 | 27.22 | **<0.01** |
| Jul | 0.24 ±0.04 | 0.29 ±0.02 | -4.96 | 27.73 | **<0.001** |
| Aug | 0.25 ±0.04 | 0.31 ±0.02 | -6.35 | 28.86 | **<0.001** |
| Sep | 0.26 ±0.04 | 0.33 ±0.02 | -6.57 | 29.11 | **<0.001** |
| Oct | 0.27 ±0.04 | 0.32 ±0.02 | -4.91 | 28.64 | **<0.001** |
| Nov | 0.26 ±0.04 | 0.35 ±0.02 | -8.63 | 30.41 | **<0.001** |
| Dec | 0.30 ±0.04 | 0.37 ±0.02 | -6.20 | 29.10 | **<0.001** |
| Orographic Soaring | |  |  |  |  |
| Jan | 0.27 ±0.03 | 0.26 ±0.02 | 0.63 | 33.55 | 0.53 |
| Feb | 0.27 ±0.03 | 0.26 ±0.02 | 1.89 | 33.37 | 0.07 |
| Mar | 0.28 ±0.03 | 0.27 ±0.02 | 1.09 | 33.48 | 0.28 |
| Apr | 0.28 ±0.03 | 0.28 ±0.02 | 0.17 | 33.62 | 0.87 |
| May | 0.29 ±0.03 | 0.28 ±0.02 | 0.68 | 33.35 | 0.50 |
| Jun | 0.29 ±0.03 | 0.30 ±0.02 | -0.57 | 33.55 | 0.57 |
| Jul | 0.29 ±0.03 | 0.29 ±0.02 | 0.66 | 33.34 | 0.51 |
| Aug | 0.29 ±0.03 | 0.28 ±0.02 | 1.18 | 33.39 | 0.25 |
| Sep | 0.29 ±0.03 | 0.28 ±0.02 | 1.21 | 33.36 | 0.23 |
| Oct | 0.28 ±0.03 | 0.28 ±0.02 | 0.11 | 33.64 | 0.91 |
| Nov | 0.29 ±0.03 | 0.27 ±0.02 | 1.58 | 33.49 | 0.12 |
| Dec | 0.27 ±0.03 | 0.26 ±0.02 | 0.91 | 33.50 | 0.37 |
| Total Soaring | |  |  |  |  |
| Jan | 0.58 ±0.04 | 0.64 ±0.02 | -5.82 | 22.80 | **<0.001** |
| Feb | 0.57 ±0.04 | 0.64 ±0.02 | -7.61 | 23.25 | **<0.001** |
| Mar | 0.56 ±0.04 | 0.62 ±0.02 | -5.92 | 22.78 | **<0.001** |
| Apr | 0.55 ±0.04 | 0.60 ±0.01 | -5.21 | 22.62 | **<0.001** |
| May | 0.54 ±0.04 | 0.59 ±0.01 | -4.75 | 22.73 | **<0.001** |
| Jun | 0.54 ±0.04 | 0.58 ±0.01 | -3.65 | 22.66 | **<0.01** |
| Jul | 0.54 ±0.04 | 0.58 ±0.01 | -4.40 | 22.78 | **<0.001** |
| Aug | 0.54 ±0.04 | 0.60 ±0.01 | -5.48 | 22.82 | **<0.001** |
| Sep | 0.55 ±0.04 | 0.61 ±0.01 | -5.84 | 22.83 | **<0.001** |
| Oct | 0.55 ±0.04 | 0.61 ±0.01 | -5.04 | 22.66 | **<0.001** |
| Nov | 0.55 ±0.04 | 0.62 ±0.02 | -7.76 | 23.11 | **<0.001** |
| Dec | 0.57 ±0.04 | 0.64 ±0.02 | -6.00 | 22.77 | **<0.001** |

Table S7. Summary of average (Temperature and wind speed are mean. Wind direction is mode) monthly weather variables from 100 randomly sampled actual weather scenarios per month (from 2012-2013) recorded by the South African Weather Services at Lambert’s Bay (32.0350S, 18.3320E) for the Sandveld and Clanwilliam (32.1760S, 18.8880E) for the Cederberg. Abbreviations: Phase = breeding phase, where br = during the breeding season, and non = out side of the breeding season for Verreaux’s eagles.

|  |  | Temperature °C | | Wind speed ms^-1^ | | Wind direction | |
| --- | --- | --- | --- | --- | --- | --- | --- |
| Month | Phase | Cederberg | Sandveld | Cederberg | Sandveld | Cederberg | Sandveld |
| Jan | non | 30.2 | 22.3 | 2.7 | 3.7 | N | W |
| Feb | non | 28.7 | 21.5 | 2.6 | 3.4 | N | W |
| Mar | non | 27.6 | 21.3 | 2.4 | 3.4 | S | W |
| Apr | non | 24.2 | 19.6 | 1.4 | 3.3 | N | S |
| May | br | 18.8 | 16.3 | 1.3 | 2.6 | N | N |
| Jun | br | 14.8 | 14.5 | 1.1 | 3.3 | N | N |
| Jul | br | 15.2 | 14.4 | 1.4 | 3.0 | N | N |
| Aug | br | 14.9 | 13.7 | 1.6 | 3.0 | N | N |
| Sep | br | 17.8 | 15.6 | 2.0 | 3.3 | N | S |
| Oct | br | 23.0 | 17.9 | 2.6 | 4.1 | S | S |
| Nov | non | 26.1 | 20.2 | 3.0 | 3.8 | S | S |
| Dec | non | 30.5 | 21.3 | 2.6 | 3.4 | N | W |
| Average | br | 17.4 | 15.4 | 1.6 | 3.2 | N | S |
| Average | non | 27.9 | 21.0 | 2.5 | 3.5 | N | W |


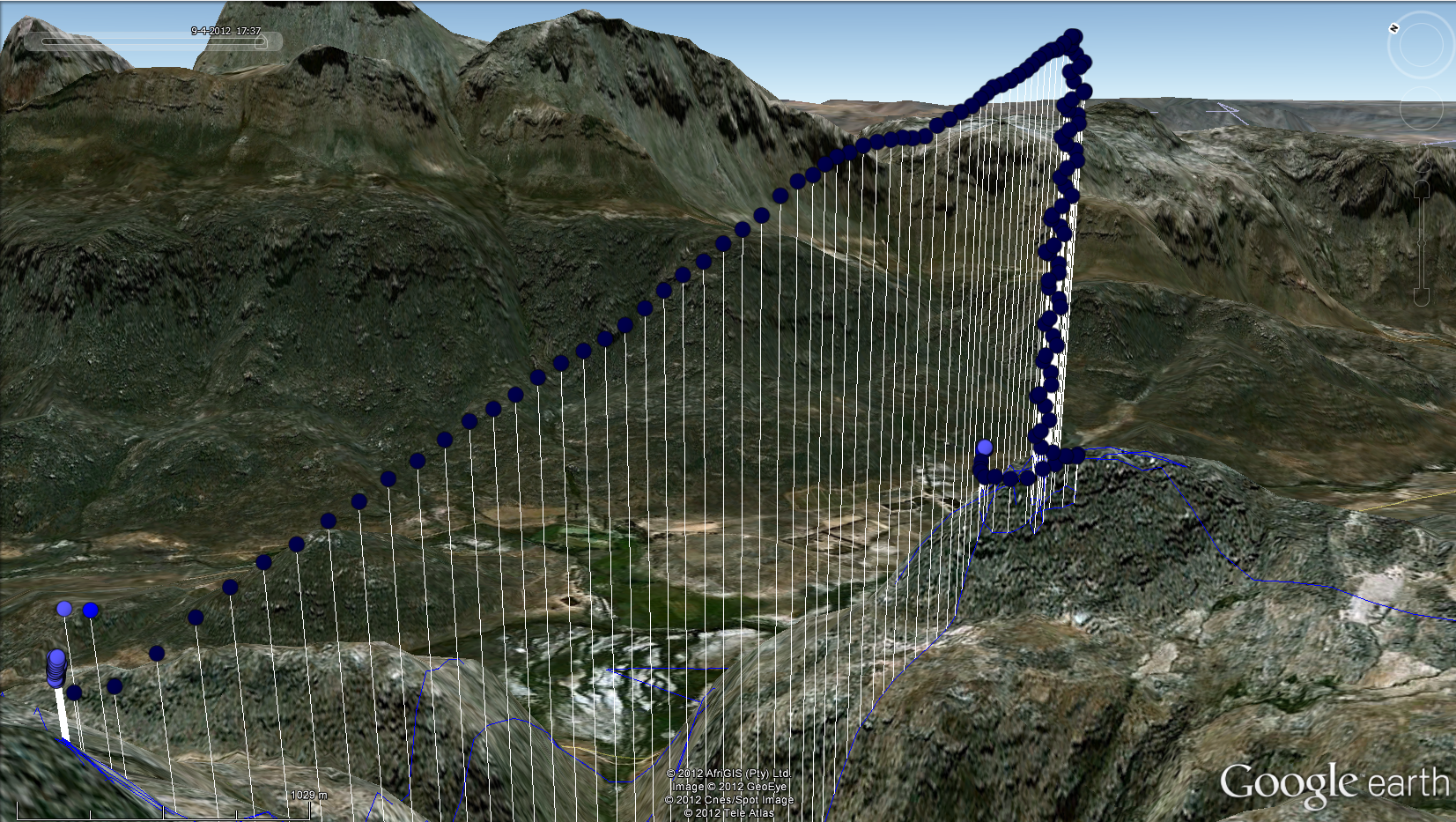
Figure S1. Representation of three minutes worth of high-resolution GPS data depicting a Verreaux’s eagle increasing more than 300m in altitude by thermalling, followed by gliding over 3.5 km across a valley (Google Earth).


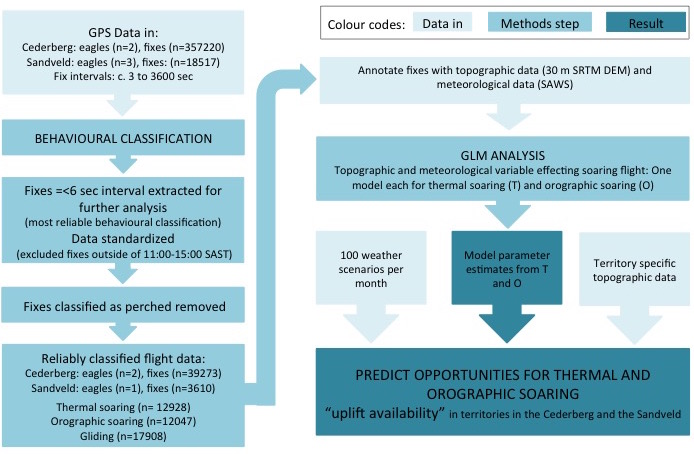
Figure S2. Flow chart showing steps described in methods for understanding flight behaviour of Verreaux’s eagles. Abbreviations used: SRTM DEM; 30 m-resolution Shuttle Radar Topography Mission digital elevation model. SAWS; South African Weather Services, this data was recorded every hour at two nearby (*c.*30 km) weather stations.


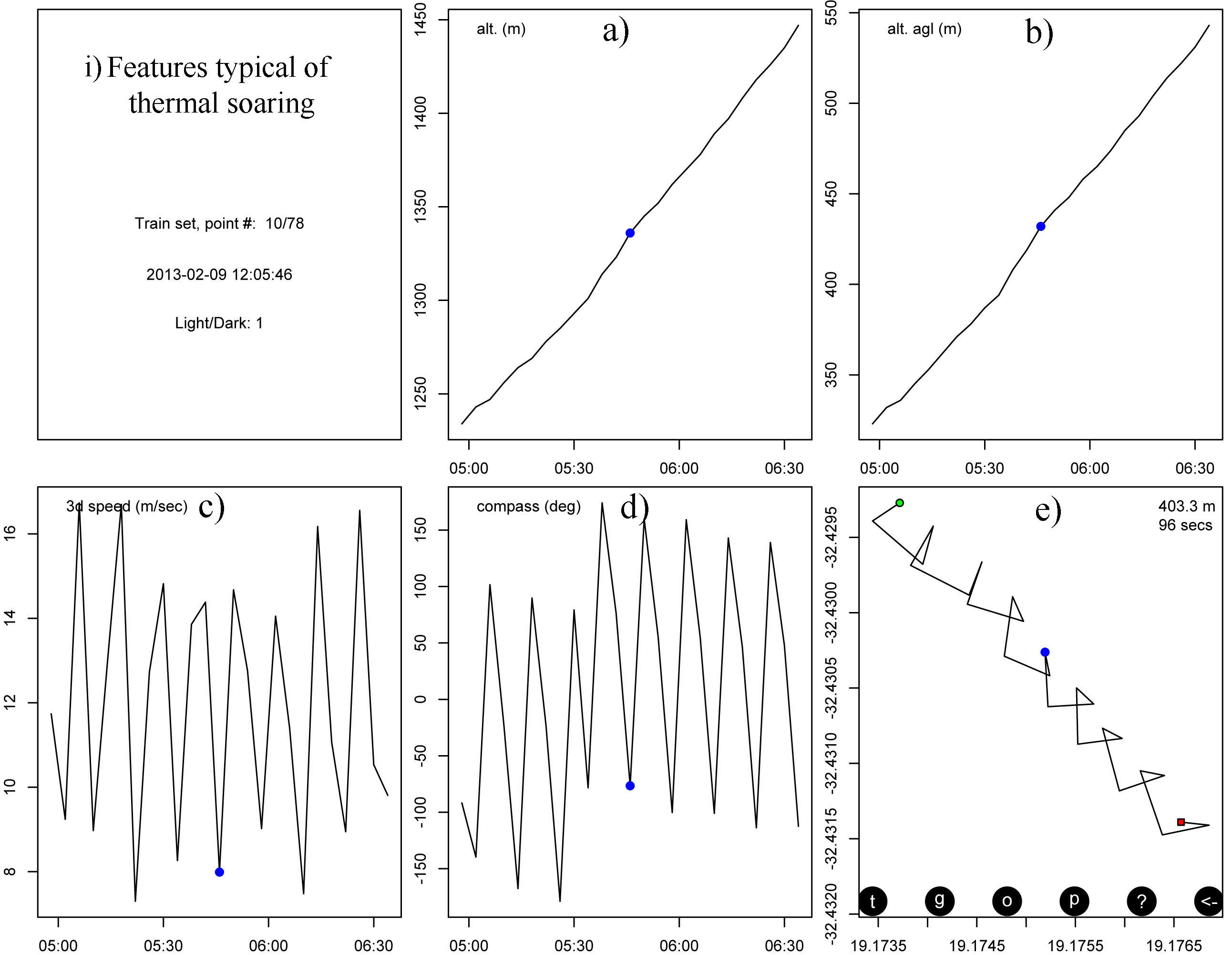

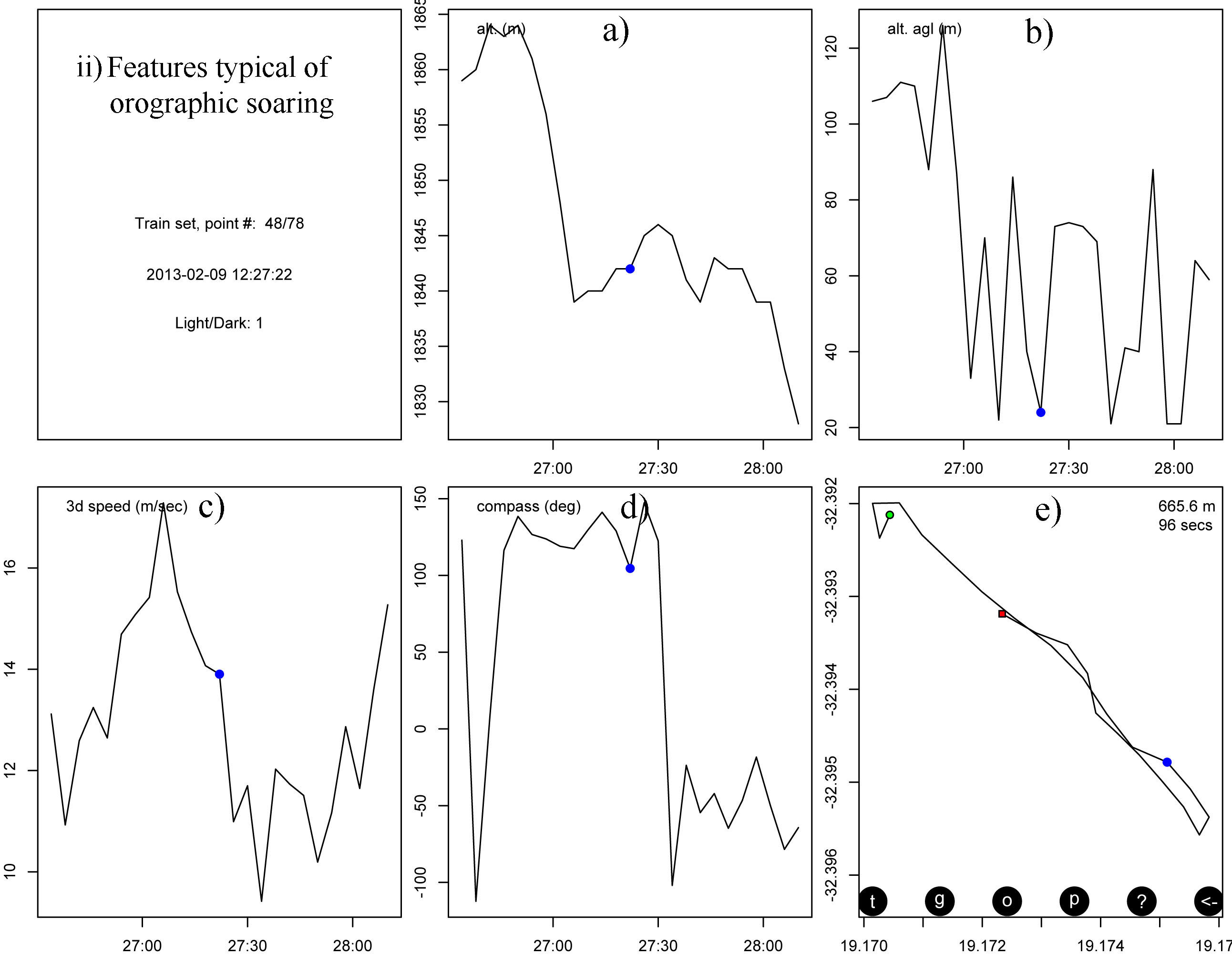


Figure S3 (continued next page).


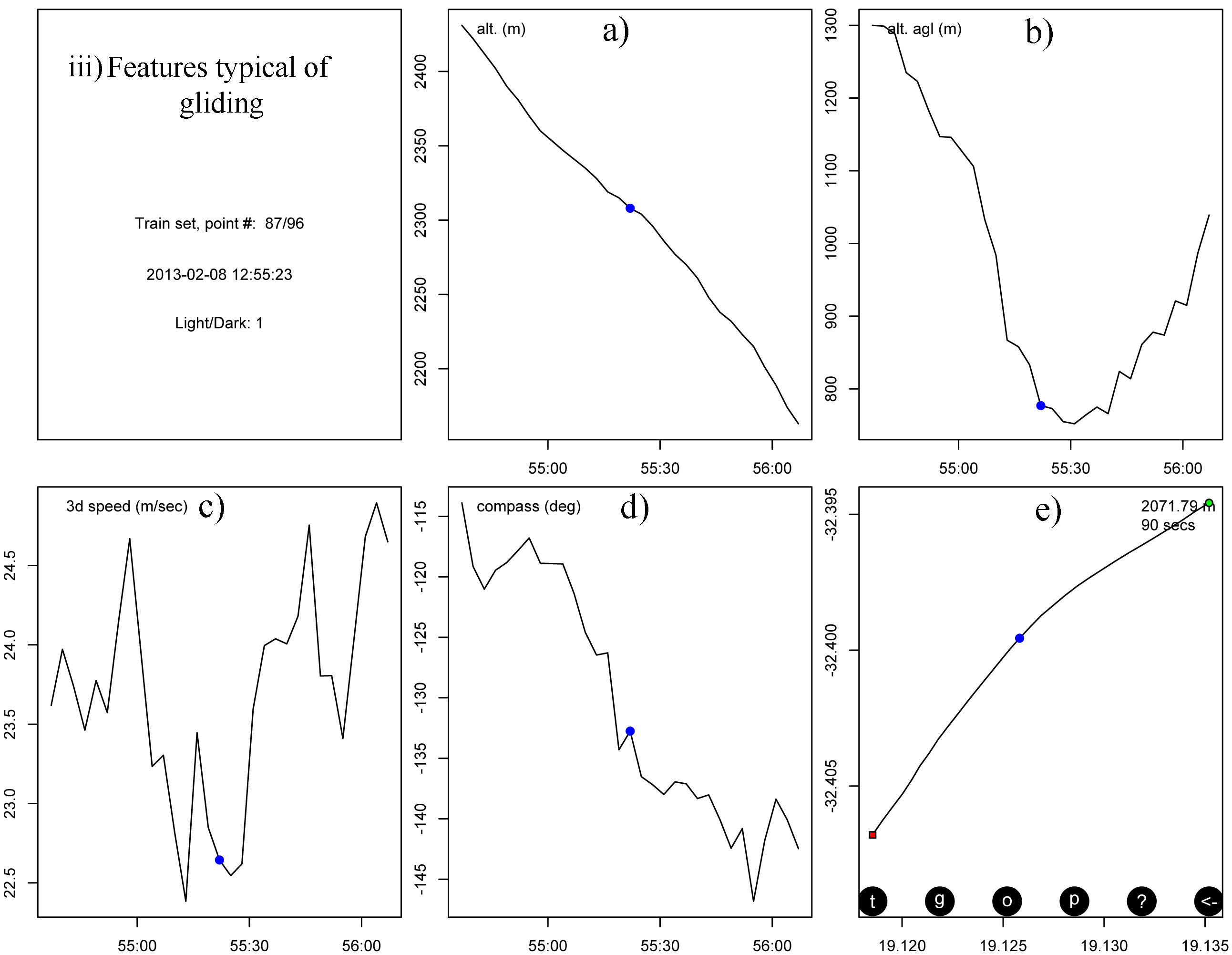

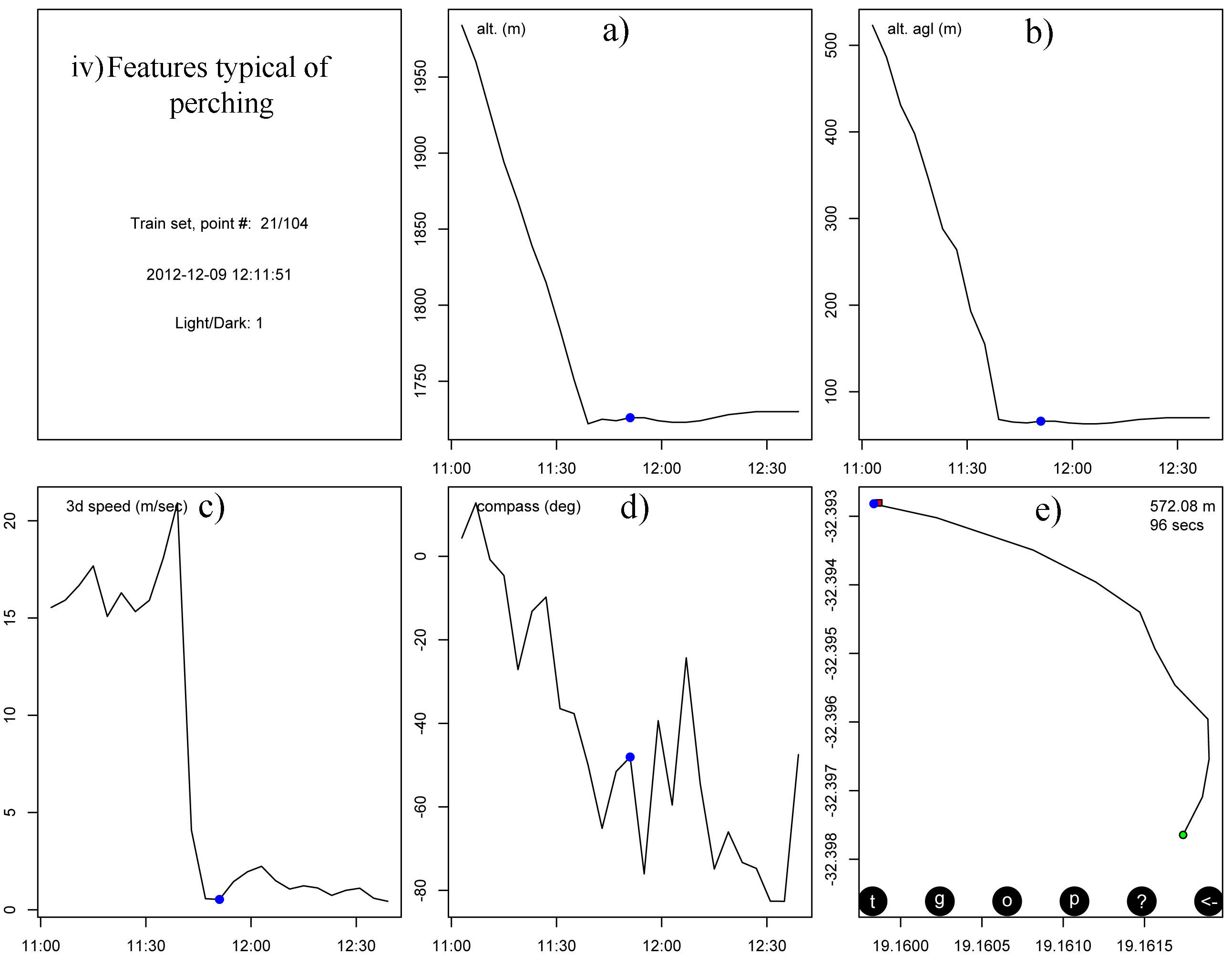


Figure S3. This figure is made up of four screenshots. Each one (i to iv) shows the plots generated by the custom-written R function (R Core Team 2016) used to manually train the random forest algorithm for classifying flight behaviour. Each set of five figures (a to e) shows the features extracted from an example track segment (90 sec) from high-resolution Verreaux’s eagle GPS data. These segments illustrate the signals that are typical of the following behaviours: i) thermal soaring; ii) orographic soaring; iii) gliding flight; and iv) perching. Movement features which are extracted from Verreaux’s eagle tracking data to identify behaviour within the segment include: a) altitude above sea level over time; b) altitude above ground level over time; c) three-dimensional instantaneous speed over time; d) the series of compass bearings over time and e) latitude and longitude of the track segment, including duration and spatial extent of the segment in terms of distance travelled (top right corner). The lettered bubbles at the bottom of plot e) are the clickable flight behaviours that made it possible to quickly and easily manually label a point based on what is shown in the five plots. In all plots the blue dot denotes the point being classified, in plot e) the green dot denotes the beginning of the track segment and the red square denotes the end of the track.

Figure S3 i) shows the typical features of thermal soaring flight. Specifically, steady gain in altitude above sea level (plot a) and ground level (plot b), cyclical changes in three-dimensional speed (plot c), as well as cyclical changes in compass bearing (plot d), and the characteristic spiral flight pattern (plot e).

Figure S3 ii) shows the typical features of orographic soaring flight. Specifically, variable gain in altitude above sea level (plot a) and ground level (plot b), variable and irregular three-dimensional speed (plot c), variable and irregular compass bearing (plot d), and flight along a narrow “corridor” (plot e).

Figure S3 iii) shows the typical features of orographic soaring flight. Specifically, steady loss of altitude about sea level (plot a) and variable change in altitude above ground level (plot b), variable but quite high three-dimensional speed (plot c), fairly steady compass bearing consistent with directed flight (plot d) and a straight and directed flight trajectory (plot e).

Figure S3 iv) shows the typical features of perching behaviour. Specifically, almost no change in altitude above sea or ground level (plots a and b), consistently low three-dimensional speed (plot c) variable and irregular compass bearing (plot d) and little or no horizontal movement (plot e).


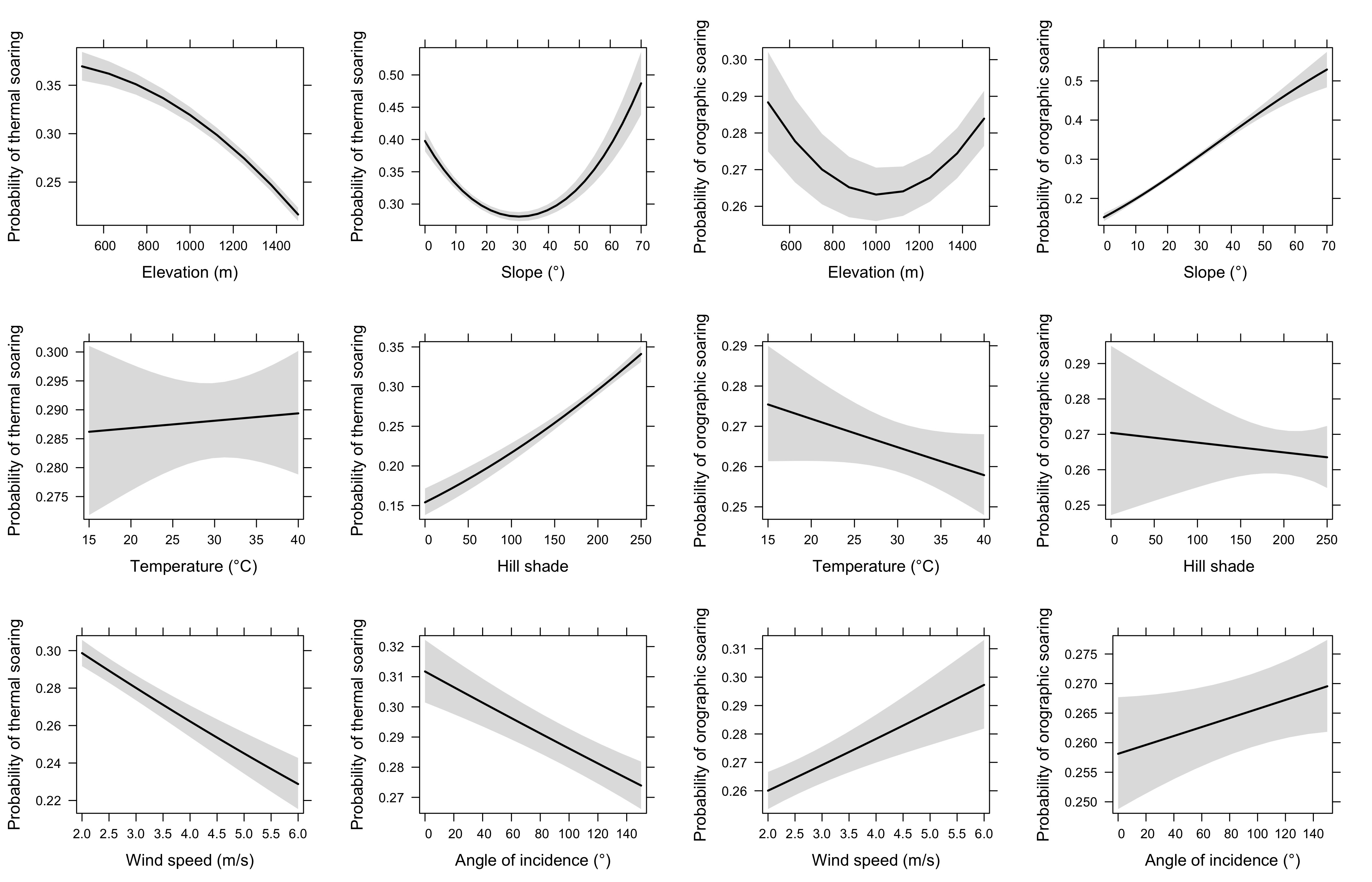


Figure S4. The effects of topographic (elevation, slope,) and meteorological (temperature, wind speed) and combined variables (angle of incidence between wind direction and topographic aspect and hill shade which represents sun exposure on slopes) on soaring probability (thermal and orographic soaring) by Verreaux’s eagles.

Appendix S1. This is an R function for angle of incidence (aoi, *v*) between the topographic aspect and wind direction. Values can range from zero to 180°, whereby high values correspond with the wind directly hitting the slope (e.g., a south facing slope and wind coming from the south). *v* equal to 90° would be equivalent to wind perpendicular to the topographic aspect (e.g. a south facing slope and wind coming from either the east or the west). Lowest values correspond with the lowest potential to generate orographic uplift, where the wind is coming from directly behind a slope. Input data are the aspect of the slope (aspect, in degrees) and the wind direction (wd, in degrees).

aoi= function(aspect, wd){

m=aspect-wd

b=ifelse(m>180, m-360, m)

n=ifelse(b<(-180), b+360, b)

v1=abs(n)

v=180-v1

return(v)

}
